# Distinct Circadian Assessments From Wearable Data Reveal Social Distancing Promoted Internal Desynchrony Between Circadian Markers

**DOI:** 10.3389/fdgth.2021.727504

**Published:** 2021-11-16

**Authors:** Yitong Huang, Caleb Mayer, Olivia J. Walch, Clark Bowman, Srijan Sen, Cathy Goldstein, Jonathan Tyler, Daniel B. Forger

**Affiliations:** ^1^Department of Mathematics, Dartmouth College, Hanover, NH, United States; ^2^Department of Mathematics, University of Michigan, Ann Arbor, MI, United States; ^3^Department of Neurology, University of Michigan, Ann Arbor, MI, United States; ^4^Department of Mathematics and Statistics, Hamilton College, Clinton, NY, United States; ^5^Michigan Neuroscience Institute, University of Michigan, Ann Arbor, MI, United States; ^6^Department of Computational Medicine and Bioinformatics, University of Michigan, Ann Arbor, MI, United States; ^7^Division of Pediatric Hematology/Oncology, Department of Pediatrics, University of Michigan, Ann Arbor, MI, United States; ^8^Michigan Institute for Data Science, University of Michigan, Ann Arbor, MI, United States

**Keywords:** circadian rhythm, wearables, internal desynchrony, heart rate, social distancing

## Abstract

Mobile measures of human circadian rhythms (CR) are needed in the age of chronotherapy. Two wearable measures of CR have recently been validated: one that uses heart rate to extract circadian rhythms that originate in the sinoatrial node of the heart, and another that uses activity to predict the laboratory gold standard and central circadian pacemaker marker, dim light melatonin onset (DLMO). We first find that the heart rate markers of normal real-world individuals align with laboratory DLMO measurements when we account for heart rate phase error. Next, we expand upon previous work that has examined sleep patterns or chronotypes during the COVID-19 lockdown by studying the effects of social distancing on circadian rhythms. In particular, using data collected from the Social Rhythms app, a mobile application where individuals upload their wearable data and receive reports on their circadian rhythms, we compared the two circadian phase estimates before and after social distancing. Interestingly, we found that the lockdown had different effects on the two ambulatory measurements. Before the lockdown, the two measures aligned, as predicted by laboratory data. After the lockdown, when circadian timekeeping signals were blunted, these measures diverged in 70% of subjects (with circadian rhythms in heart rate, or CRHR, becoming delayed). Thus, while either approach can measure circadian rhythms, both are needed to understand internal desynchrony. We also argue that interventions may be needed in future lockdowns to better align separate circadian rhythms in the body.

## Introduction

Until recently, assessment of circadian rhythms was restricted to laboratory studies. In these studies, individuals needed to isolate themselves from external Zeitgebers such as light, and researchers had to monitor physiological signals such as the onset of melatonin secretion (DLMO) over intervals ranging from hours to almost 2 days (1–4). Two recent techniques have been developed which now allow researchers to supplement these typical intensive experiments through mobile assessment of circadian rhythms using wearables (5, 6). Wearables typically collect data on wrist movement (actigraphy) and heart rate, each of which can separately be used to estimate various outputs of the circadian clock in the body (5–11). Using mathematical models and activity measurements collected by the Apple Watch, Huang et al. recently showed that DLMO can be predicted to within ~1 h in non-shift workers (5), which is much more accurate (in terms of mean absolute error between a laboratory DLMO measurement and predicted phase) than cosinor analysis of ambulatory circadian rest activity [the average difference between in laboratory DLMO and the acrophase from cosinor analysis of rest-activity cycle is 4.47 and 4.6 h from Huang et al. (5) and Woelders et al. (12), respectively]. Similar results have been found with other data sets across a variety of studies (12–14). Alternatively, Bowman et al. studied the circadian rhythm in heart rate (CRHR), which originates not in the brain but the sino-atrial (SA) node of the heart (6, 15). With a Bayesian framework, they isolated the intrinsic circadian phase of heart rate and determined the phase uncertainty of this marker. Interestingly, Bowman et al. were also able to determine differences in intrinsic circadian timekeeping between individuals (6). These two methodologies provide powerful tools for the assessment of circadian rhythms in the field.

The 2020 COVID-19 lockdowns were one of the largest global changes in human behavior. These lockdowns had measurable effects on the timing of sleep, meals, and other activities, which mainly shifted to later times of the day (16–22). Social pressures on the timing of daily activities greatly changed, with many businesses and schools operating remotely or closing. Many individuals restricted their light exposure to indoor light, which is less efficient at entraining circadian rhythms (23–27). They also increased their exposure to computers, smartphones, and tablets before bedtime (28, 29), which are known to disrupt circadian rhythms (30), and adjusted the timing of meals, which can shift the timing of clocks in peripheral tissues but not the central clock in the brain (31). Thus, we wondered how measures of human circadian rhythms changed in the presence of the altered timing of key entraining signals (Zeitgebers).

To collect wearable data to study the effects of the lockdowns on measures of circadian rhythms, in June 2020 we launched the Social Rhythms app [http://umich.edu/~socialrhythms/], which remains available on the iTunes App and Google Play stores (6). Individuals upload heart rate and activity data collected by a wearable device from a user-specified period. Users then receive a report showing how their circadian rhythms changed after social distancing. All data and reports that are sent to or from the app's servers are transmitted anonymously. Analysis of this rich wearable data set using the methods of Huang et al. and Bowman et al. could help determine how social distancing and the 2020 COVID-19 lockdowns affected circadian rhythms (5, 6).

Here, we first compare the estimated circadian phase of heart rate with actual laboratory measurements of DLMO collected as part of a previous study (32). Prior to DLMO collection, individuals lived outside of the lab and were not given any specific instructions about how or how often to use the Apple Watch. Even so, we found that 8 out of 10 DLMO measurements were within the 80% confidence intervals of the heart rate circadian phase, indicating a degree of alignment between the circadian rhythms in the heart and the brain under normal circumstances. We then carefully looked at the profiles of activity and heart rate collected by the Social Rhythms app. A variety of schedules were present in the data set, including 4 college students, 18 parents, and 1 shift worker. Nevertheless, 78% of the DLMO predictions are within the predicted 80% confidence intervals of the estimated heart rate rhythm. Surprisingly, this alignment dropped in 70% of individuals (including 61% of the parents and 100% of the college students selected in the analysis) after the onset of social distancing. From this, we suggest that social distancing and the COVID lockdowns increased the internal desynchrony between markers of peripheral circadian rhythms. Importantly, our results set the stage for using the emerging paradigm of coupling rigorous mathematical modeling and wearable data to assess circadian phase in individuals in the field setting.

## Materials and Methods

### DLMO Data Set

Dim light melatonin onset (DLMO) is the gold-standard phase marker in the field of human circadian rhythms. We recruited subjects living in Michigan for laboratory DLMO measurements, each of whom was given an Apple Watch to wear in the preceding weeks (32). In total, 20 subjects exhibited melatonin onset during the study (i.e., had a valid DLMO measurement). Although individuals were recommended to wear the watch for most of each day, the use of the watch varies and we did not require subjects to wear it for a specific length of each day. Due to the inherent charging requirements of the Apple Watch, gaps of six or more hours are commonly present in the wearable portions of this data set and often coincide with periods of sleep. Considering the accuracy and ability to simulate the mathematical model, 10 subjects who missed wearing the watch for more than 1 day were excluded in our following analysis, and it left us 10 subjects who had at least 7 days of consecutive heart rate and activity data. It is worth noting that the Apple Watch reported data in uneven intervals. Thus, heart rate and activity data were averaged into 5-min bins to avoid the effect of disproportionate sampling.

### Social Rhythms Data Set

Researchers at the University of Michigan developed the Social Rhythms Application, a mobile app that utilizes data from phones and wearable devices in order to provide feedback to the users about the state and features of their personal circadian clock. Of 201 subjects submitted from worldwide (165 from America, 15 from Europe, 13 from Asia, 5 from Australia, and 3 from Pacific time zone), 135 subjects who had steps and/or heart rate data with a self-reported social distancing date were extracted for data analysis (122 from America, 7 from Europe, 4 from Asia, 1 from Australia, and 1 from Pacific time zone). In particular, model simulations and data averages used a subset containing 72 subjects (63 from America, 3 from Europe, 4 from Asia, and 2 from Pacific time zone), who submitted both daily motion and heart rate data (i.e., wearable devices were worn everyday) during the entire period of 70 days (35 days before and after social distancing). Of these subjects included in the analysis, 18 were parents, 4 were college students, and 1 was a shift worker. The data set considered here consisted of steps and heart rate measurements collected from two widely available devices, Apple Watch and Fitbit. In particular, heart rate and activity data were resampled into 5-min intervals to account for the effect of disproportionate measurements. Besides physiological data, users voluntarily submitted self-reported demographic information, such as age, gender, living condition, and the time when they started social distancing. The Social Rhythms app collects all data anonymously and sends back reports anonymously. The University of Michigan IRB determined that the use of this data was not regulated. Users can choose how much data to send and can remove data from the Social Rhythms servers. Users submitted data between June 10th and August 19th, 2020, and had the option to submit retrospective data from before these dates. Average steps and heart rate data profiles in this data set were calculated by taking the mean activity and heart rate in 5-min bins (as in the Bayesian algorithm), and then averaging over all the days.

### Bayesian Algorithm

To analyze the circadian phase in heart rate, we adopt the Bayesian approach from Bowman et al. (6). It assumes that heart rate consists of a 24 h periodic oscillation, plus a separate term to account for the effect of exercise on heart rate. This yields a model for heart rate:


(1) $$\begin{array}{l} HR\;=\;a\;-\;b\;\cdot \cos\left(\frac{\pi }{12}\;\left(Time\;-\;c\right)\right)+d\;\cdot Activity\;+\;\epsilon . \end{array}$$

Here, *a* denotes the basal heart rate, *b* is the amplitude of the circadian rhythm of heart rate, *c* is the time (in hours) at which the circadian minimum of heart rate occurs, *d* is the change in heart rate per unit of activity, and ϵ is the error of the model. This error term follows the autoregression AR (1) error model, and it can be further broken down as $\epsilon_{t+1}\;=\;k\cdot \epsilon_{t}\;+\;N\left(0,\sigma^{2}\right)$. That is, a fraction *k* of the noise at time t is carried over at time t + 1, and σ represents independent measurement error or new external effects. The values for *HR, Time*, and *Activity* come from the wearable data set. For each individual, the six parameters of the model are fitted directly from the data by using Goodman and Weare's affine-invariant Markov chain Monte Carlo algorithm, a likelihood-based approach that provides error estimates (33). The previous day's fit is used as a prior distribution to predict the successive days, and this enables the computation of both mean estimates and uncertainties for the parameters on a daily basis. Specifically, parameters were fitted by testing 100,000 probabilistically chosen samples for each day of data and the burn-in ratio was set as 0.5. More details of the method can be found in Bowman et al. (6) (and the codes to run this algorithm are openly available at https://github.com/pepperhuang/heartrate).

### Limit-Cycle Model

We apply a limit-cycle oscillator model of the human circadian clock to predict the circadian phase marker of DLMO. This model describes the effect of light on the human circadian pacemaker (34):


(2) $$\dot{x}=\frac{\pi }{12}\left[x_{c}+\mu \left(\frac{1}{3}x+\frac{4}{3}x^{3}-\frac{256}{105}x^{7}\right)+B\right]$$

(3) $$\begin{array}{l} \overset{∙}{x_{c}}=\;\frac{\pi }{12}\left\{qBx_{c}-\left[{\left(\frac{24}{0.99729\tau_{x}}\right)}^{2}+kB\right]x\right\} \end{array}$$

(4) $$\dot{n}=60\left[\alpha \left(1-n\right)-\text{ β}n\;\right],$$

where


(5) $$\begin{array}{l} \mu \;=0.13,\;B=\left(1-0.4x\right)\left(1-0.4x_{c}\right)\hat{B},\\ \text{​​​​​ }\hat{B}=G\alpha \left(1-n\right),\;\alpha =\alpha_{0}{\left(\frac{I}{I_{0}}\right)}^{p},\\ \;G\;=19.875,\;\alpha_{0}=0.16,\;I_{0}=9500, \end{array}$$

*p* = 0.6, τ_*x*_ = 24.2, *k* = 0.55, and β = 0.013. The first two equations describe a limit-cycle oscillator for *x* and *x*_*c*_, where the variable *x* reflects the core body temperature rhythm and *x*_*c*_is a mathematically required complementary variable to achieve the limit cycle.

This model has been validated against multiple carefully controlled laboratory studies (35, 36), and light level (in lux) is the only input in the original design of the model. However, recent advances in the field have shown that incorporating activity is necessary to predict the circadian phase in the field setting (5, 12, 37). In particular, activity is correlated with light, and it has been shown that replacing light by activity levels measured from wearable devices coupled with the mathematical model can predict circadian phase with an error within 1 h for people living in regular conditions (5, 12), Initial conditions were generated from a limit-cycle [as in Huang et al. (5)]: the start point was determined to be 35 days prior to the social distancing date, and the model was simulated until 35 days after social distancing. See Huang et al. (5) for further details of the model and the implementation. The codes for model implementations are available at https://github.com/pepperhuang/predictCircadianRhythms.

## Results

**Figure 1:**
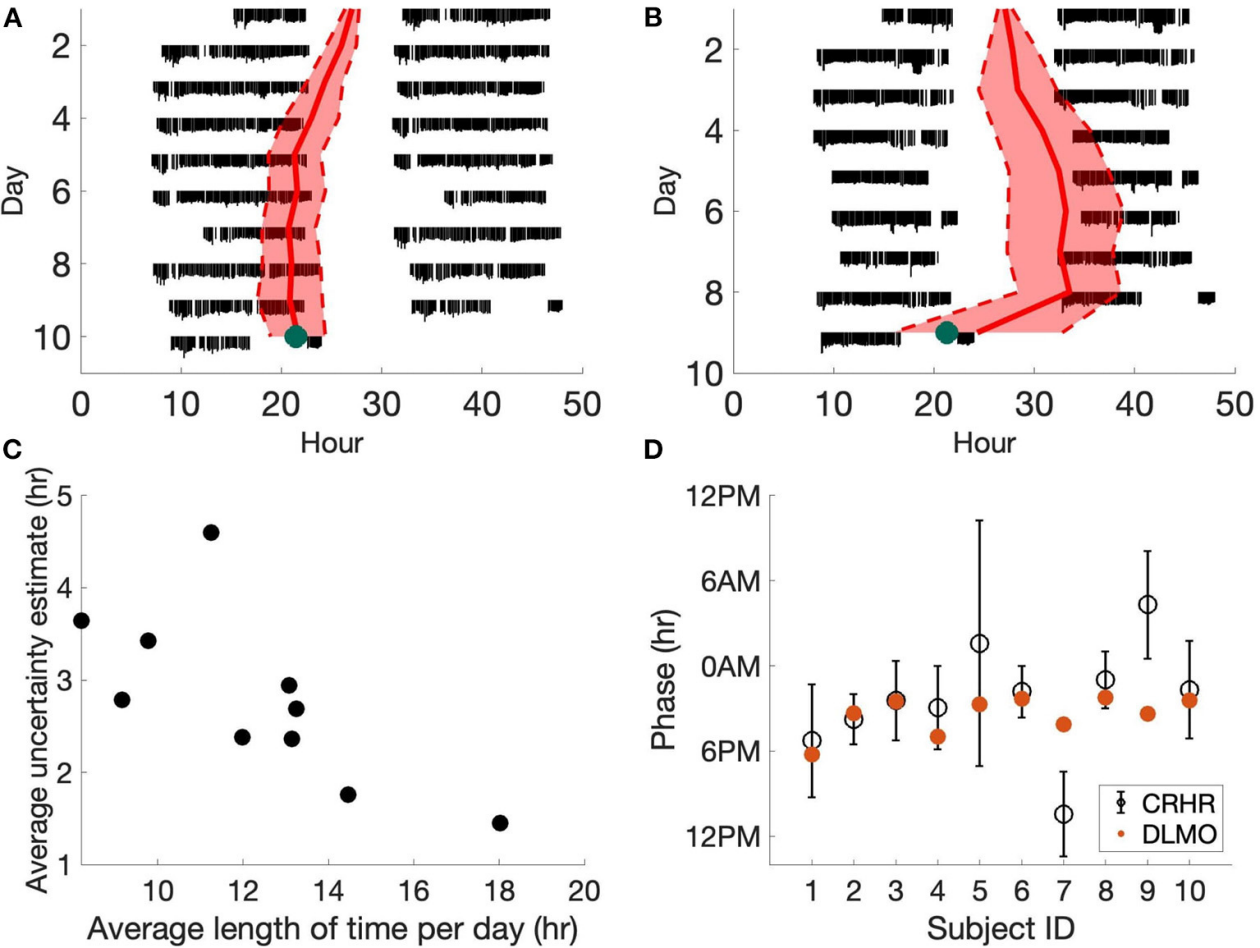
Heart rate phase and DLMO. **(A,B)** Actograms for two subjects generated from Apple Watch data. Estimated CRHR phase (red, with 80% confidence bands) is overlaid on daily heart rate patterns (black). Laboratory DLMO measurement is marked by the green dot. The subject in **(A)** wore the device for longer during the day than the subject in **(B)** which led to more accurate circadian phase measurements. **(C)** For each subject, we computed the average length of time wearing the device per day. We can see that the uncertainty estimates of the phase of the CRHR tend to decrease as the user wears the watch for longer periods of the day. **(D)** Estimated CRHR phase (black circle, with 80% confidence bands) are plotted for all ten subjects, where red dots denote laboratory DLMO measurements. CRHR phases in **(A,B,D)** were shifted by −4.4 h to account for the mean difference between DLMO and CRHR.

We first compare the phase estimates of the CRHR to clinical measures of the phase of the central circadian pacemaker (i.e., DLMO). It is important to note this study uses data from the Apple Watch, which we showed has larger phase uncertainty (and thus may be less accurate in predicting circadian phase) than other devices (6). Additionally, individuals in this cohort were not required to wear the watch continuously. For these reasons, error estimates from this dataset are larger than our previous study. Figure 1 shows two sample subjects from the DLMO data set cohort. The magnitude of heart rate measurements is shown in black with separate days plotted vertically. Data are double plotted to show patterns, as is typical for actograms. The daily predicted phase of the CRHR is shown by the red line, plotted with the 80% confidence interval in these measurements. On the final day, the DLMO measurement is shown by the green dot (Figures 1A,B).

When comparing the heart rate and melatonin rhythms from the DLMO data set, we found that 8 out of 10 subjects had a DLMO measurement within the 80% confidence intervals of the phase of CRHR, when CRHR is shifted by a fixed constant of −4.4 h (Figure 1D) to account for the average phase difference between these markers across the population. This shows that circadian rhythms in the central pacemaker and heart tend to be synchronized during normal conditions.

We also note one additional trend. The uncertainty estimates of the phase of the CRHR tend to increase as the user wears the watch for shorter periods of the day (Figure 1C). For the ten subjects from the DLMO data set, we found a statistically significant negative relationship between the length of time wearing the device and the uncertainty estimate (*p* = 0.018). In fact, a similar result was also discovered in the Social Rhythms data set, where the correlation between the number of data points and the phase uncertainty in the Social Rhythms data set is −0.40 (*p* = 9.73e-271). This further corroborates a significant negative correlation between the number of data points and the uncertainty estimate.

**Figure 2:**
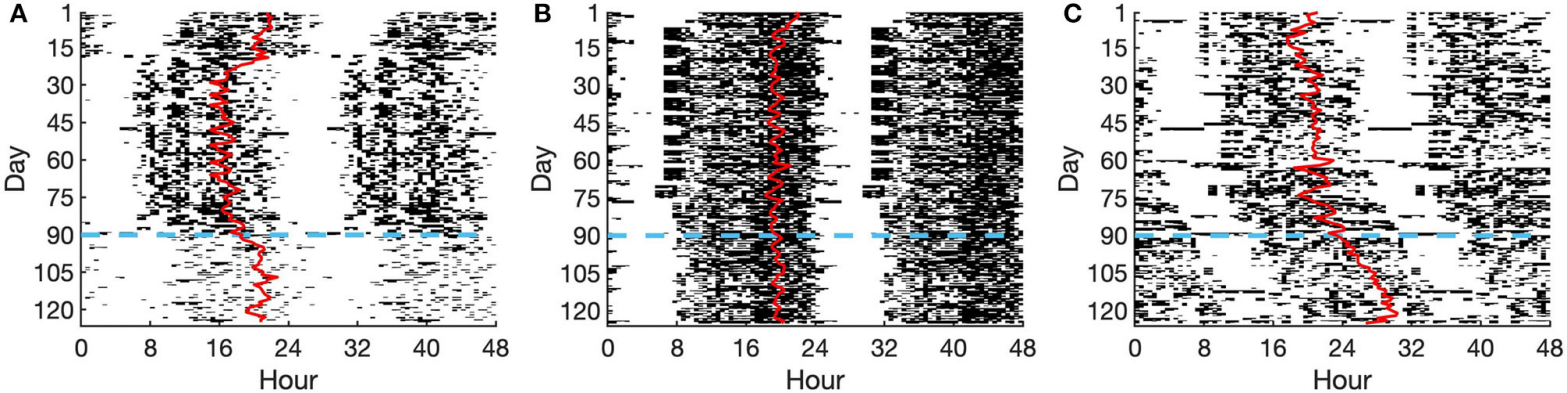
Actograms of selected individuals. **(A)** College student, **(B)** Parent, **(C)** Shift worker. Activity is shown as dark bars, with thicker bars corresponding to more steps taken at that time. The red line represents the DLMO phase prediction from the limit-cycle oscillator model, and the horizontal dashed blue line denotes the date of social distancing onset.

We next investigated how social disruptions affect the alignment between the CRHR and the melatonin rhythm. The COVID-19 social distancing and lockdowns presented a unique opportunity to test whether these rhythms become desynchronized in the wake of societal and/or behavioral changes. To study this, we used data from the Social Rhythms app, through which individuals reported when they began social distancing and uploaded their wearable data. In total, 72 subjects uploaded both motion and heart rate data within 35 days before and after a self-reported social distancing start date. Three sample actograms are shown in Figure 2, representing a college student (Figure 2A), a parent (Figure 2B), and a shift worker (Figure 2C). The day when social distancing began is shown in blue (Figure 2).

**Figure 3:**
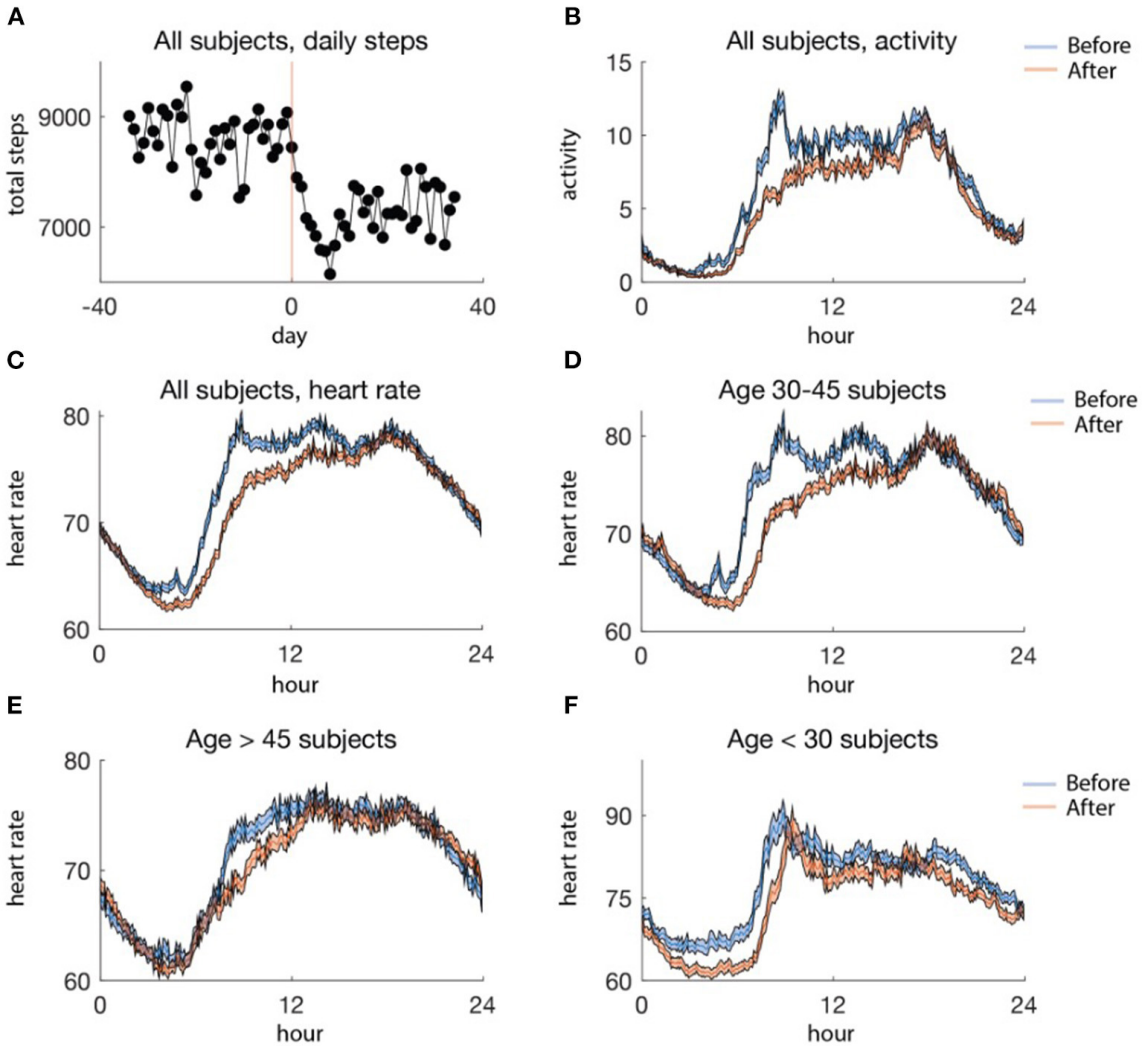
**(A)** Average aggregate steps per day, aligned at the social distancing date. The red line (day 0) is the date of social distancing onset, and the black dots are the mean steps per day. **(B)** Activity profile, averaged by 5 min for data before (blue) and after (red) social distancing. **(C)** Average daily heart rate profiles for all subjects binned in 5-min intervals, before (blue) and after (red) social distancing. **(D–F)** Average heart rate profiles binned in 5-min intervals by age group, before (blue) and after (red) social distancing. **(D)** includes data from 33 subjects, **(E)** includes data from 26 subjects, and **(F)** includes 13 subjects. In **(B–F)** the standard error of the mean is shaded.

We examined how wearable activity and heart rate data changed after social distancing in Figure 3. The amount of activity decreased by about 18%, on average across the population, after social distancing (Figure 3A). Moreover, average daily activity decreased as a function of the time of day. In particular, there was a steeper decrease in activity earlier in the day rather than later, until the very end of the day, when trailed off earlier after social distancing than before (Figure 3B). This is in line with previous studies which have reported increased sleep during the pandemic. No clear patterns were seen separating the data into age groups.

In contrast to activity, there was no clear pattern in the average raw heart rate data from before and after social distancing in the whole population (Figure 3C). However, when we separated out into age groups, a pattern could be observed (Figures 3D–F). In particular, individuals younger than 30 and aged between 30 and 45 tended to have a later increase in heart rate in the morning after social distancing (Figures 3D,F) perhaps due to changed AM responsibilities. Moreover, an earlier decrease in heart rate in the evening after social distancing was observed in the group of individuals below 30 (Figure 3F). However, such trends are not necessarily indicative of changes in the circadian phase and could be due to other factors such as activity.

**Figure 4:**
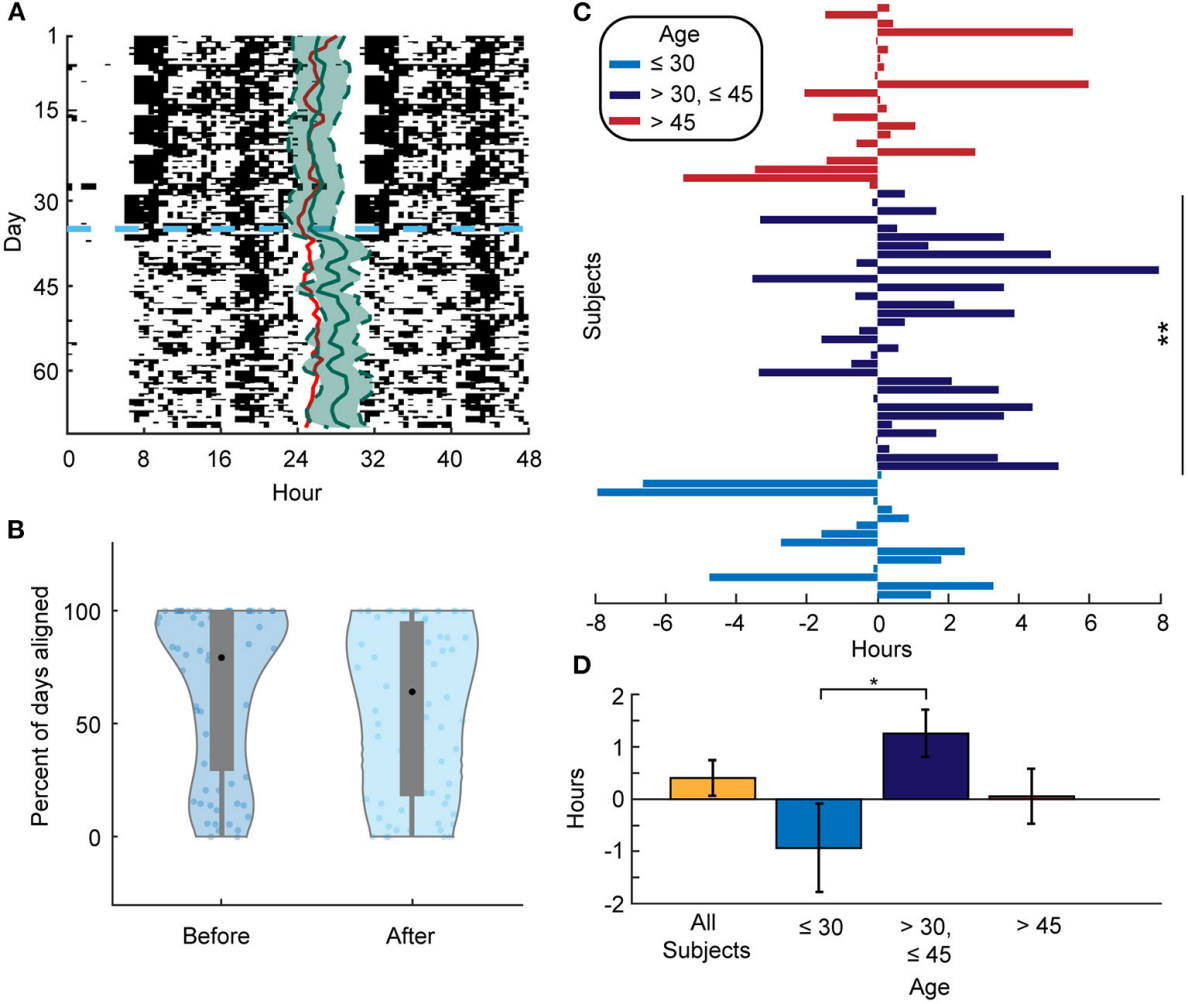
**(A)** Actogram for a selected individual. The red line represents the DLMO phase prediction from the limit-cycle oscillator model, and the horizontal dashed blue line denotes the date of social distancing onset. Estimated CRHR phase (solid green line) with 80% confidence interval (dashed green line) is overlaid on daily activity patterns. As in Figure 1, CRHR phases were shifted by −4.4 h to adjust for the mean difference between DLMO and CRHR. **(B)** Violin plots showing the distribution of the percent of days that an individual's DLMO estimate was within the heart rate phase estimate +/– the 80% confidence interval both before (left) and after (right) social distancing. The mean percentage before is 78.08 and 69.39% after (*p* = 0.002). **(C)** Phase differences for all subjects from before and after social distancing centered for each individual at the mean phase in the 35 days before social distancing. A positive (negative) value means that the mean phase after social distancing was later (earlier) than the mean phase before for that individual. Subjects between 30 and 45 years of age had a significant phase shift later (*p* = 0.0089). **means *p*-value ≤ 0.01. **(D)** Mean phase difference (phase estimate after social distancing–phase estimate before social distancing) for the respective population (All subjects: 0.25 +/– 0.34 h, < 30 years of age: −0.93 +/– 0.85 h, 30–45 years of age: 1.26 +/– 0.45 h, and > 45 years of age: 0.05 +/– 0.53 h). The phase differences of the under 30 and the between 30 and 45 years of age groups are significantly different (*p* = 0.016). *denotes level 0.05 of statistical significance.

We next examined the alignment between the predicted DLMO phase and the estimated CRHR. We found that 78% of the predicted DLMO timings from the validated methods of Huang et al. (5) were within the 80% confidence interval of the CRHR phase. This matches the results shown in Figure 1 and provides additional evidence that separate circadian rhythms in the body are aligned under normal circumstances. On the other hand, the majority of individuals (51 out of 72) showed less alignment between these separate phase markers after social distancing, when entraining signals are likely shifted or reduced (one example presented in Figure 4A). In particular, all four college students included in the data analysis showed less alignment after social distancing, and 11 out of 18 parents exhibited more misalignment as well. Moreover, the mean percentage of days of alignment before social distancing was 78% as opposed to 69% of days after social distancing (*p* = 0.002) (Figure 4B). In addition, the mean difference between estimated CRHR and DLMO increased from 4.61 to 5.58 h after social distancing, which further reflects less alignment between these two markers when lockdown was imposed.

We did not see significant phase shifts across social distancing onset in the predicted DLMO between age groups. However, we did detect these differences in CRHR phase estimates. In particular, younger individuals (<30 years of age) tended to keep an earlier CRHR post-distancing, whereas individuals aged between 30 and 45 averaged a later CRHR (Figures 4C,D). In fact, the average phase difference from before to after social distancing is 1.25 h in the 30 to 45 age group but −0.93 h in the group <30 years of age (*p* = 0.016, Figure 4D).

## Discussion

Our study provides the first real-world measurements of circadian rhythms during the pandemic. However, as wearables provide multiple potential markers of human circadian rhythms, an important outstanding question is when these markers align. Our results suggest that in normally entrained scenarios, the two markers of CRHR and DLMO exhibit alignment. Previous work, in the absence of available heart rate data, shows that the predicted DLMO from a limit-cycle oscillator model may be a viable marker (5, 12). However, actigraphy is the only input for the limit-cycle oscillator model, and this model will not account for certain variations in the intrinsic circadian timekeeping system between individuals. These effects, for example interindividual differences in the intrinsic circadian period and sensitivity to light, are known to impact human circadian rhythms (1, 38). Such differences are not included in the limit-cycle model, however it does seem unlikely that these differences could create the systematic differences we observe. In scenarios in which these variations may be particularly relevant, the CRHR phase estimate may be the best choice, since parameters are fitted to each individual. However, when traveling across time zones, working on shifts, or in cases where Zeitgebers may be weak or ambivalent, the combination of both markers may be best suited to determine potential internal circadian desynchrony. Several studies have highlighted this desynchrony as a major health consequence (39).

Our basic hypothesis is that the lockdowns dramatically changed zeitgebers, and these changes in circadian signaling affect different markers differently. We found that two measures of circadian rhythms predicted by mathematical models became desynchronized after social distancing. Significant phase shifts were observed in CRHR estimates before and after social distancing between different age groups. However, no significant phase shifts across social distancing onset was found in the predicted DLMO. This can be explained by two possibilities. One possibility is that the model framework is different for the two measures, DLMO and CRHR. As discussed above, the CRHR algorithm might pick up some signals that the limit-cycle model does not. In addition, seasonal differences may also have affected the measures of the circadian rhythms (40). Therefore, it is necessary to develop personalized models of the human circadian clock to account for the effect of interindividual differences in the future. The other explanation is that CRHR measures more closely follow the peripheral clocks, but DLMO measures are more closely aligned to the central circadian pacemaker. Thus, CRHR estimates shifted significantly during the lockdown, since external cues, such as meals and social interaction, have a larger effect on peripheral clocks (31, 41–43). Previous work has also shown that alterations to sleep patterns occur during the pandemic (16–22). However, our CRHR algorithms do not consider data during sleep, and are not simply a reflection of changed sleep-wake patterns [See (6)]. Future work is needed to address whether the changes in circadian markers were due to decreased exposure to external light, changes in activity or meals, or actual social distancing (i.e., being physically separated from other human beings).

The source of the various signals driving circadian timekeeping systems remains to be explored. It is highly likely that external light exposure, the primary stimulus to the human circadian pacemaker, exhibited differently during the lockdown. Future work should explore to what extent electric light and daylight affect circadian rhythms during COVID-19. Another possibility is that mealtime shifted during the pandemic: this matches the results to some degree, since meals are known to shift the CRHR but not the DLMO clock. Additionally, over 50% of individuals under 30 years old lived with parents during the pandemic, the largest percentage on record (44). Such living arrangements could explain why younger individuals had an earlier CRHR phase. However, individuals older than 45 did not show the opposite trend. Finally, individuals between 30 and 45 years old shifted later. This could be due to changes in work habits or not having to bring children to school early in the morning. However, we did not have enough individuals in our data set to significantly parse out these trends.

The data examined here are particularly challenging for the CRHR algorithm since users were not instructed on exactly how long to wear the device or what kind of schedule to lead. Thus, these data should represent normal casual use by individuals not necessarily in a study. Indeed, we found a significant negative correlation between the quantity of data and the phase uncertainty in both the DLMO data set and the Social Rhythms data set. This suggests that future studies should consider instructing individuals to wear the Apple Watch for as long as possible during the waking day. As the current study does not impose these constraints and thus presents perhaps the most difficult possible scenario for the CRHR algorithm, the results gave us confidence that we could use the algorithm with wearable data that had been collected by the watch in the field (and not as part of any controlled study). Additionally, as shown in Bowman et al. estimates of CRHR from the user available Apple Watches tend to have larger errors than those from other devices (e.g., Fitbits), since far fewer HR measurements are available (6). However, we note that the device could take more measurements if it were changed from its normal settings. These findings could therefore help in the design of future studies.

Limitations of the predicted DLMO methods and the CRHR phase estimates are discussed in Huang et al. (5) and Bowman et al. (6), respectively. We do note that some scenarios can bias these estimates. For example, individuals with very different circadian timekeeping systems from normal (or shift workers) may be difficult for the DLMO prediction methods to accurately assess. Although the limit-cycle model has been validated in papers for over 20 years against DLMO, including a recent paper with several wearable data sets (5), actual DLMO measurements would yield stronger results. Likewise, certain activities, for example, particular kinds of exercise or pharmacological agents, could alter heart rate in ways that the methods of Bowman et al. (6) cannot remove. Finally, although encouraging, our results are based on a relatively small sample of subjects. The relationship between CRHR and DLMO should be further explored in a larger data set, with more variations in demographic information such as age. In particular, more middle-aged subjects (30–45 years old) participated in the Social Rhythms app, which might contribute to the statistical significance found in this age group. Therefore, larger data sets need to be analyzed to discover further demographic differences in the effects of social distancing.

## Data Availability Statement

The raw data was collected under the terms of Social Rhythms Privacy Policy, which allows the summary data to be shared. The summary data presented in the study is deposited in https://github.com/pepperhuang/socialrhythms_frontiers_digital_health. Other raw data used in the study can be found in (5).

## Ethics Statement

The studies involving human participants were reviewed and approved by University of Michigan IRB. The patients/participants provided their written informed consent to participate in this study.

## Author Contributions

YH, CM, OW, CG, JT, and DF collected data. YH, CM, JT, and DF analyzed the data. YH, CM, OW, CB, SS,CG, JT, and DF discussed the study and wrote the manuscript. All authors contributed to the article and approved the submitted version.

## Funding

This work was supported by the following Grant Nos. NIMH 101459, HFSP RGP 0019/2018, and NSF 1714094 and 2052499.

## Conflict of Interest

OW has given talks at Unilever events and received honorariums/travel expenses. She is the CEO of Arcascope, a company that makes circadian rhythms software. DF is the CSO of Arcascope. They and the University of Michigan are part owners of Arcascope. Arcascope did not sponsor this research. CG receives royalties from UpToDate. The remaining authors declare that the research was conducted in the absence of any commercial or financial relationships that could be construed as a potential conflict of interest.

## Publisher's Note

All claims expressed in this article are solely those of the authors and do not necessarily represent those of their affiliated organizations, or those of the publisher, the editors and the reviewers. Any product that may be evaluated in this article, or claim that may be made by its manufacturer, is not guaranteed or endorsed by the publisher.
